# When algorithms evaluate people: how AI video interviews relate to employability confidence and organizational attractiveness

**DOI:** 10.3389/fpsyg.2026.1927892

**Published:** 2026-09-25

**Authors:** Weice Shen, Meng Gao

**Affiliations:** Soochow University, Suzhou, China

**Keywords:** algorithmic transparency, organizational attractiveness, perceived employability, PLS-SEM, situated expectancy-value theory

## Abstract

Algorithmic evaluation is increasingly entering high-stakes social contexts, including recruitment, where young people are assessed before any human interaction occurs. AI video interviews are one such setting; they are largely one-directional, opaque, and often provide no feedback about how applicants are evaluated. This study examines how such AI-mediated evaluation relates to two socially consequential perceptions among university students: confidence in their own employability and trust-related evaluations of organizations that deploy AI-based assessment. Drawing on Situated Expectancy-Value Theory (SEVT), we distinguish between expectancy beliefs—whether one can succeed—and value beliefs—whether the outcome is worth pursuing, and develop a dual-pathway model in which expectancy-related individual factors (AI literacy, metacognition, need for cognition, and technology anxiety) are associated with perceived employability, while value-related process perceptions (procedural justice and algorithmic transparency) are associated with organizational attractiveness, with social comparison orientation and AI usage experience as boundary conditions. Using data from 520 Chinese university students analyzed with PLS-SEM, we find that all four expectancy-related factors are independently associated with perceived employability, and that procedural justice and algorithmic transparency are each substantially associated with organizational attractiveness. AI usage experience and social comparison orientation further moderate the hypothesized relationships. The findings contribute to debates on AI-mediated evaluation, algorithmic transparency, and the social consequences of using AI in early career transitions.

## 1 Introduction

AI video interviews have rapidly emerged as a standard screening tool in graduate recruitment. Industry surveys indicate that AI adoption in hiring nearly doubled in a single year, rising from 26 to 43% of organizations worldwide (SHRM, 2025), and an estimated 99% of Fortune 500 companies now incorporate some form of AI into their recruitment processes (Bogen and Rieke, 2018). In these interviews, applicants record responses to standardized prompts while an AI system analyzes verbal content, vocal features, and facial expressions to generate assessments (Hickman et al., 2022; Langer et al., 2019). Many university students are encountering formal job interviews for the first time—this algorithmic evaluation often constitutes the first contact with a prospective employer, one that occurs before any human interaction. Unlike traditional interviews, where candidates can read interpersonal cues and adapt in real time, AI video interviews are largely one-directional: evaluation criteria are often opaque, feedback is absent, and the basis of assessment is unclear (Langer et al., 2018; Nørskov et al., 2020). This opacity creates a dual challenge for students’ career development. First, without clear evaluation standards, applicants may struggle to assess their own readiness, which could undermine their confidence in their employability—a subjective self-appraisal that depends in part on the availability of performance-relevant information (Rothwell and Arnold, 2007; Vanhercke et al., 2014). Second, without sufficient understanding of what the algorithm values, applicants cannot judge whether the selection process is fair—and because applicants routinely use selection procedures as signals of organizational attributes (Rynes et al., 1991; Gilliland, 1993), this uncertainty may weaken organizational attractiveness (Chapman et al., 2005).

The rise of AI video interviews reflects a broader societal shift in which algorithmic systems increasingly participate in consequential evaluations of individuals. Such systems do not merely assist organizations in screening candidates; they also shape how people understand themselves, their opportunities, and the institutions that evaluate them. For students entering the labor market, an AI interview may be more than a selection procedure. It may become an early encounter with algorithmic authority, influencing both their confidence in navigating future employment and their willingness to trust organizations that rely on opaque AI systems. Both educational institutions and the research community have responded to these challenges. That attention has produced a substantial and increasingly precise body of work. Applicants react less favourably to asynchronous video interviews than to synchronous ones, and their standing relative to other digital preselection instruments has been compared directly (Basch et al., 2022; Dunlop et al., 2025). The design features intended to improve those reactions have been mapped in detail, with effects that are often limited (Lukacik and Bourdage, 2025): the tangibility, immediacy and transparency of the AI interface bear on trust in the system (Suen and Hung, 2023), avatar characteristics bear on interactional justice perceptions (Min et al., 2024), and the explanations accompanying a rejection bear on organizational attraction indirectly, through procedural justice and interpersonal treatment (Falls et al., 2025). Transparency has been examined both as a general property of algorithm-based selection (Langer and König, 2023) and as a lever on applicant responses, though these effects are not always straightforward (Lakhiwal et al., 2026; Niessen and Neumann, 2026). A parallel line has established that the evaluation itself is substantive rather than nominal: automated competency assessments derived from video interviews show measurable psychometric properties across organizational samples (Liff et al., 2024), and AI systems can extract behavioural signals fine enough to distinguish deceptive from honest impression management (Hung et al., 2026). Applicants respond accordingly. The presence of AI assessment and the interface through which it is delivered are associated with how candidates present themselves, altering both honest and deceptive impression management in field settings (Suen and Hung, 2024, 2025), and applicants’ willingness to be evaluated algorithmically depends on how they expect to fare under it (Liang et al., 2025).

Taken together, this work indicates that an AI video interview is a consequential algorithmic evaluation environment rather than another digital interview format. What an applicant faces is a system that observes multiple aspects of their behaviour, scores them on criteria they cannot see, and does so with enough fidelity that their conduct changes in anticipation of it. An encounter of that kind is unlikely to leave only one impression. It carries information about the organization that chose to evaluate this way, and it carries information about the applicant, about how they perform when the evaluator is opaque and the interpersonal cues they would ordinarily read are absent. The applicant-reactions literature has developed the first of these carefully. The second, and the question of whether the two arise together and share antecedents, has received less attention: how is being evaluated by AI related to applicants’ perceptions of themselves and of the institutions that evaluate them? This question is increasingly important because algorithmic evaluation is no longer confined to organizational decision-making. Instead, it has become a social experience through which individuals form beliefs about their future opportunities and about the legitimacy of organizations that deploy AI systems. In response to the growing prevalence of AI-mediated assessment (Long and Magerko, 2020; Ng et al., 2021; Shi and Wang, 2025), both practitioners and researchers have sought to identify factors that may help individuals navigate algorithmic evaluation more effectively. This work falls into two largely separate streams. One, centered on the applicant, treats the individual’s own competencies as the key to navigating AI assessment, showing that AI literacy improves preparedness (Wang et al., 2023) and that practice with AI mock interviews strengthens graduates’ career identity and confidence (Shi and Wang, 2025; Bennett and Ananthram, 2022). The other, centered on the organization, treats the properties of the selection process as decisive, showing that the fairness and transparency of AI systems shape how candidates regard the hiring organization (Langer et al., 2019; Ochmann et al., 2024; Köchling et al., 2023). The two streams speak to two different questions an applicant faces—can I succeed at this, and is the organization behind it worth pursuing—yet they have developed in isolation, and each has largely stopped at applicants’ immediate reactions, such as anxiety and fairness perceptions, rather than the judgments that carry into the school-to-work transition (Woods et al., 2020). Where the two have been brought into contact, it has been on the organizational side of the ledger: Falls et al. (2025) trace how rejection explanations reach organizational attraction through justice perceptions, and Liang et al. (2025) show that applicants’ acceptance of algorithmic decision-making turns on the advantage they expect it to give them. What the applicant concludes about their own standing from the same encounter, and whether that conclusion is reached through the same features of the process, is not addressed by either. The deeper limitation is that these two questions are handled by two literatures, when in the AI interview they arise from a single encounter. A traditional interview already served two functions at once: it evaluated the applicant and, through the interviewer’s conduct, conveyed what the organization was like (Dipboye, 1992; Rynes et al., 1991; Chapman et al., 2005). An AI interview removes the interpersonal exchange but not these two functions; it collapses them onto one opaque agent that is, at the same moment, the evaluator of the applicant and the source of the applicant’s impression of the organization. The applicant thus leaves with two judgments formed from the same event: an expectancy judgment about whether they can succeed, and a value judgment about whether joining is worth pursuing. This is the distinction Situated Expectancy-Value Theory is built on, which makes it a useful framework here: SEVT treats achievement-related choices as the joint product of expectancy and value, and so lets us ask whether the two judgments an applicant leaves with are associated with different antecedents, and how far each is specific to its own—questions neither literature has been positioned to answer. When it comes to sustaining willingness to join organizations that use AI selection, is procedural fairness more consequential than algorithmic transparency, or are both equally critical? Answering them requires modeling both judgments together within the expectancy–value framework and comparing the relative weight of their antecedents.

We propose that Situated Expectancy-Value Theory (SEVT; Eccles and Wigfield, 2020) provides such a framework. SEVT posits that achievement-related actions are jointly determined by individuals’ expectations of success and the value they attach to the outcome. These two components function simultaneously; when either falls short, action is undermined even if the other is high. In the AI interview, both evaluative processes are engaged at once. The expectancy-related process is the applicant’s appraisal of whether they can perform in this format; the value-related process is their appraisal of whether an employer that screens this way is worth pursuing. Neither process is confined in its consequences to the interview itself. Performing under algorithmic evaluation is a test case for a form of assessment applicants expect to meet again, so what the expectancy process yields feeds a standing judgment about their employment prospects: perceived employability, a broader confidence about obtaining and maintaining work. What the value process yields likewise feeds an evaluation of the particular employer that chose to screen this way, its attractiveness. These two judgments are where the processes register, not the processes themselves. We use SEVT to organize the two processes and to specify what each draws on. The expectancy process is fundamentally inward-directed (Eccles and Wigfield, 2020; Wigfield and Eccles, 2000), a judgment about the self and one’s own capacity, and should therefore be most strongly shaped by the cognitive, metacognitive, and affective resources that students bring to the encounter (Bandura, 1997). The value process concerns the worth, to the student, of pursuing this organization—a judgment they infer from what the selection procedure reveals about how the organization regards them (Eccles and Wigfield, 2020; Rynes et al., 1991; Turban, 2001)—and should therefore be most strongly shaped by observable features of how the AI selection process is conducted. Accordingly, we develop a dual-pathway model in which four individual factors (AI literacy, metacognition, need for cognition, and technology anxiety) predict perceived employability through the expectancy pathway, and two process perceptions (procedural justice and algorithmic transparency) predict organizational attractiveness through the value pathway, with social comparison orientation and AI usage experience as boundary conditions. We test this model with 520 Chinese university students (Woods et al., 2020; Langer et al., 2019), a group for whom AI video interviews are becoming a routine part of the transition from education to employment. More specifically, we address three research questions: (1) Are expectancy-related individual factors and value-related process perceptions associated with perceived employability and organizational attractiveness in the differentiated way the two processes imply? (2) Within each pathway, does each antecedent contribute independently of the others? (3) Do AI usage experience and social comparison orientation qualify these associations?

Our research contributes to the extant literature in four ways. First, this study contributes to AI and society research by conceptualizing AI video interviews as a form of algorithmic evaluation rather than merely a recruitment technology (Shi and Wang, 2025). In doing so, we show that AI-based interviews are not only tools for organizational screening, but also consequential social encounters through which young people experience algorithmic authority during the transition from education to work. Second, by applying Situated Expectancy–Value Theory to AI-mediated evaluation, this study distinguishes two partially differentiated forms of perception: how individuals evaluate themselves and how they evaluate the organizations that deploy AI. The expectancy-related process registers in students’ confidence in their own employability; the value-related process registers in their evaluation of the organization as fair, transparent, and worth pursuing. Third, the cross-path results show where the two sets of perceptions overlap and where they do not. How applicants read the procedure, whether it was fair and whether it was intelligible, was unrelated to how capable they judged themselves to be: admitting both process perceptions into the employability equation raised explained variance by less than 0.001. Variance in self-appraisal was accounted for by what applicants brought to the encounter rather than by how they perceived the process itself, and three of the four resources that accounted for it are not specific to AI. Policy discussion treats readiness for algorithmic selection largely as a matter of AI literacy (Long and Magerko, 2020; Ng et al., 2021; World Economic Forum, 2025); these results place most of the weight elsewhere. They also separate two levers that are usually discussed together. Perceived transparency was associated with how applicants viewed the employer, but not with how capable they judged themselves to be. Fourth, the moderation results indicate that these associations are not uniform across students: their strength varied with AI usage experience and with social comparison orientation, which suggests that preparation programs may need to be tailored to students’ prior experience and individual characteristics rather than adopting a uniform approach.

## 2 Theoretical background and hypotheses development

### 2.1 Situated expectancy-value theory

In the recruitment and career development literature, motivation has been a central lens for understanding how students navigate the transition from education to employment. Motivational frameworks have been used to explain why students engage in career planning, how they sustain effort during job search, and what determines whether they pursue or withdraw from employment opportunities (Kanfer et al., 2001; Turban et al., 2009; Wanberg et al., 2020). Situated Expectancy-Value Theory (SEVT; Eccles and Wigfield, 2020; Eccles et al., 1983) is one of the most influential of these motivational frameworks, originally developed to explain students’ achievement-related choices in educational settings (Wigfield and Eccles, 2000) and subsequently applied to domains including STEM career choice (Eccles and Wang, 2016), academic persistence (Watt et al., 2012), and course enrollment decisions (Wigfield et al., 2015). SEVT highlights two core beliefs that jointly determine whether individuals pursue a given activity: expectations of success—whether they believe they can perform well—and subjective task value—whether they believe the outcome is worth pursuing (Eccles and Wigfield, 2020). These two components function multiplicatively: when either falls short, motivation is undermined even if the other is high (Wigfield and Eccles, 2000). Many studies have applied SEVT to explain students’ choices in academic and early career contexts (Wigfield et al., 2015; Watt et al., 2012), while limited research has examined how expectancy and value beliefs operate when students face technology-mediated, high-stakes selection. In particular, in the context of AI video interviews, when students evaluate their own readiness (an expectancy judgment) and simultaneously form impressions of the hiring organization (a value judgment), how do these two evaluations take shape, and what drives each? We therefore conceptualize our empirical model within the SEVT framework, organizing the antecedents of successful employment into an expectancy pathway and a value pathway.

When students encounter an AI video interview, SEVT suggests that students are likely to engage in two evaluative processes simultaneously. The first is an expectancy process: students assess whether they have what it takes to succeed. For example, can I handle this unfamiliar format? Do I have the skills and self-regulatory resources to perform effectively under algorithmic evaluation? This process is inward-directed—it concerns the self and one’s own capacity. What students conclude from this appraisal does not remain tied to the interview: it feeds their perceived employability, the standing confidence they hold in their ability to obtain and maintain employment (Rothwell and Arnold, 2007). Because this process is fundamentally about self-assessment, it should be most strongly shaped by the individual factors students bring to the encounter—cognitive, metacognitive, and affective factors that determine how they prepare for, manage, and reflect on the AI interview experience.

The second is a value process: students assess whether the outcome is worth pursuing. Is this organization—one that uses AI to evaluate me—a place I would want to work? Does the way this process is conducted suggest that the employer is fair, transparent, and respectful? The value process concerns the worth, to the student, of pursuing this organization—a judgment they infer from what the selection procedure reveals about how the organization regards them (Eccles and Wigfield, 2020; Rynes et al., 1991; Turban, 2001)—and should therefore be most strongly shaped by observable features of how the AI selection process is conducted. What students conclude about the worth of pursuing this employer registers in organizational attractiveness, the degree to which they regard it as a desirable place to work (Highhouse et al., 2003). Because this worth-of-joining judgment is inferred from how the applicant is treated, it should be shaped most strongly by the fairness and transparency of the AI process—the cues that signal whether the organization values them. Guided by this expectancy–value distinction, we next develop hypotheses for each pathway in turn.

### 2.2 The expectancy pathway

The expectancy process concerns students’ assessment of whether they possess the resources needed to succeed in the AI interview. In SEVT, expectancy beliefs are shaped by individuals’ self-concept of ability, past performance experiences, and perceptions of task difficulty (Eccles and Wigfield, 2020; Wigfield and Eccles, 2000). When the task in question is performing under algorithmic evaluation—a high-stakes, largely one-directional format that provides minimal feedback and no interpersonal cues (Langer et al., 2018; Nørskov et al., 2020)—students must draw primarily on the internal resources they bring to the encounter. Students with stronger individual resources have more grounds for expecting to perform well under algorithmic evaluation, and we expect this appraisal to register in their perceived employability—the standing confidence they hold in their ability to obtain and maintain employment (Rothwell and Arnold, 2007; Vanhercke et al., 2014). Perceived employability is fundamentally a self-referential judgment (De Cuyper et al., 2012): it reflects not objective labor market conditions but rather students’ subjective appraisal of their own career prospects (Forrier and Sels, 2003; Jackson and Tomlinson, 2020). Individuals with higher perceived employability demonstrate more proactive job-seeking behaviour (Yizhong et al., 2017; Fernández-Valera et al., 2020). Which resources matter is dictated by the specific demands the AI interview imposes, and four features of the format each call on a different kind of resource. Its opacity—applicants cannot see what the system evaluates—calls on AI literacy, the knowledge that reduces this uncertainty. Its absence of feedback—there are no interviewer reactions to read—calls on metacognition, the capacity to monitor and adjust one’s own performance in place of external cues. Its novelty and cognitive load call on need for cognition, the disposition governing how deeply one engages an unfamiliar, effortful task. And its technology-mediated nature calls on the management of technology anxiety, the affective response that can depress the expectation of success independently of the others. These four are not an arbitrary list: they map onto the cognitive, metacognitive, motivational, and affective resources that self-regulated performance draws on (Pintrich, 2004; Efklides, 2011), and each is tied to a defining feature of the encounter. We deliberately set aside self-efficacy and perceived control, which in the expectancy–value tradition are near-constitutive of the expectancy belief itself and would be redundant with the outcome (Eccles and Wigfield, 2020), and career adaptability, which overlaps with employability and is typically its consequence rather than its antecedent. What remains are four resources the applicant brings to the encounter, each distinct from the confidence they help produce. These four features are not idiosyncratic to this study but recur across reviews of digital and automated selection, which characterize AI-based interviews as opaque, largely one-directional and low in feedback, novel relative to conventional formats, and technology-mediated with reduced social presence (Woods et al., 2020; Langer et al., 2019, 2021; Nørskov et al., 2020; Suen and Hung, 2023).

AI literacy refers to a set of competencies that enables individuals to critically evaluate, communicate with, and effectively use AI technologies (Long and Magerko, 2020; Ng et al., 2021). It encompasses understanding the basic functions of AI, recognizing how AI applications operate in everyday and professional settings, and evaluating AI outputs critically. In the AI interview context, AI literacy captures whether students understand what types of data AI systems analyze—verbal content, vocal features, facial expressions—and what criteria may inform algorithmic evaluation (Hickman et al., 2022). From an expectancy perspective, this understanding should reduce the perceived novelty and opacity of the interview format (Langer et al., 2019): students who know what AI systems assess are better positioned to calibrate their preparation and self-presentation accordingly. Recent evidence supports this reasoning—Santos-Jaén et al. (2025) found that perceived AI literacy is positively associated with both internal and external employability perceptions among university students. Similarly, students with positive AI attitudes and higher AI literacy report lower job-seeking anxiety (Li et al., 2025) and greater confidence in managing AI-mediated career challenges. Globally, AI literacy has been positioned as a core workforce readiness competency (World Economic Forum, 2025), and universities have invested heavily in AI literacy curricula on the assumption that understanding AI is a necessary precondition for navigating AI-enabled work environments (Long and Magerko, 2020; Ng et al., 2021). We therefore hypothesize:

H1. Higher AI literacy is positively associated with perceived employability.

Metacognition refers to the awareness and regulation of one’s own cognitive processes, encompassing the ability to plan, monitor, and evaluate one’s thinking and performance (Flavell, 1979; Schraw and Dennison, 1994). Where AI literacy concerns knowledge about a specific technology, metacognition concerns a broader, domain-general capacity to manage how one learns, prepares, and adapts across situations (Veenman et al., 2006). In educational settings, metacognition has been consistently linked to academic performance, self-regulated learning, and adaptive expertise (Pintrich, 2002; Zimmerman, 2002). In the career context specifically, metacognitive skills are recognized as essential for individuals to regulate their cognition and control their decisions in uncertain and changing work environments (Zimmerman, 2002). Bennett and Ananthram (2022) argue that perceived employability is ultimately shaped within graduates’ metacognitive framework: it is the capacity to reflect on one’s own strengths and gaps that translates knowledge and skills into employment confidence. In the AI interview context, metacognition should be particularly consequential. Because AI video interviews provide no real-time feedback and no interpersonal cues (Langer et al., 2018; Nørskov et al., 2020), students cannot rely on the interviewer’s reactions to adjust their performance. Instead, they must draw on their own internal monitoring—assessing whether their responses are coherent, whether their preparation was adequate, and where they need to improve (Schraw and Dennison, 1994). This reasoning suggests that metacognition contributes to employability confidence not by providing specific AI knowledge, but by enabling students to regulate how effectively they use whatever knowledge they have. We therefore hypothesize:

H2. Higher metacognition is positively associated with perceived employability.

Need for cognition (NFC) refers to individuals’ stable tendency to engage in and enjoy effortful cognitive activities (Cacioppo and Petty, 1982; Cacioppo et al., 1996). Individuals high in NFC are intrinsically motivated to seek out, elaborate on, and make sense of complex information, independent of external incentives (Cacioppo et al., 1996). NFC is distinct from both AI literacy—which concerns domain-specific knowledge—and metacognition—which concerns the regulation of cognitive processes. Instead, NFC captures the depth and quality of engagement that individuals bring to cognitively demanding tasks (Petty et al., 2009). In career-related contexts, deeper cognitive engagement has been associated with more thorough career exploration (Stumpf et al., 1983), more deliberate career planning (Turban et al., 2009), and higher job search self-efficacy (Wanberg et al., 2020), because individuals who think more deeply about career tasks are better equipped to extract meaning from those experiences. Research also shows that students who develop strong critical thinking and problem-solving skills—hallmarks of high NFC—are more confident in their transition from education to employment (Habets et al., 2020). In the AI interview context, students high in NFC should be more likely to actively analyze the demands of the unfamiliar format, think through their responses rather than rely on rehearsed answers, and engage deeply with the preparation process. From an SEVT expectancy standpoint, NFC strengthens the expectancy process not through specific knowledge or self-regulatory skill, but through the intrinsic motivation to invest cognitive effort in the encounter—effort that should translate into greater confidence in one’s own performance. We therefore hypothesize:

H3. Higher need for cognition is positively associated with perceived employability.

Technology anxiety refers to individuals’ apprehension, discomfort, or fear when interacting with technology-based systems (Meuter et al., 2003; Venkatesh, 2000). It manifests as nervousness about using unfamiliar technologies, worry about making errors, and a tendency to avoid technology-mediated situations (Parasuraman, 2000). Technology anxiety is conceptually distinct from the three resources discussed above: where AI literacy concerns what students know, metacognition concerns how they regulate their thinking, and NFC concerns how deeply they engage, technology anxiety concerns how they feel when confronted with a technology-based evaluation. It is an affective response rather than a cognitive resource (Meuter et al., 2003). Research consistently shows that technology anxiety inhibits individuals’ willingness to use and engage with technology-based services (Meuter et al., 2003; Parasuraman, 2000), reduces self-efficacy in technology-mediated tasks (Compeau and Higgins, 1995; Venkatesh, 2000), and increases avoidance behaviour in digital environments (Lemay et al., 2018). In career-related contexts, students’ orientations toward AI have been linked to job-seeking anxiety: among Chinese university students, AI attitudes and AI literacy mediated the association between career self-efficacy and job-seeking anxiety (Li et al., 2025). From an SEVT perspective, technology anxiety undermines the expectancy process. Anxious students are likely to perceive the AI interview as more difficult and less controllable (Eccles and Wigfield, 2020), which lowers their expectation of success regardless of the knowledge, self-regulatory skills, or cognitive motivation they possess. In the AI video interview context, this is particularly consequential. The absence of real-time feedback and interpersonal cues (Langer et al., 2018; Nørskov et al., 2020) already creates an evaluative environment with high uncertainty; for students who are anxious about the technology itself, this uncertainty is compounded—they must manage not only the content demands of the interview but also the emotional burden of interacting with an unfamiliar and anxiety-provoking system. This dual load consumes cognitive resources that would otherwise be available for self-presentation and performance (Eysenck et al., 2007), further eroding their confidence in their own employability. Technology anxiety thus represents an affective barrier that can undermine the expectancy process even in the presence of relevant knowledge and self-regulatory capacity. We therefore hypothesize:

H4. Higher technology anxiety is negatively associated with perceived employability.

### 2.3 The value pathway

The value process concerns students’ assessment of whether the hiring organization is worth pursuing. In SEVT, subjective task value encompasses several components—including the intrinsic interest, utility, and personal importance of an activity, as well as its perceived costs (Eccles and Wigfield, 2020; Wigfield and Eccles, 2000). Applied to the AI interview, the activity being valued is the student’s own decision to pursue and, if selected, to join this organization—an achievement-related choice of the kind SEVT is designed to explain. The question is therefore not how the employer scores on some external checklist, but whether joining is worth the student’s investment: whether this organization regards and respects them enough to make pursuing it worthwhile. Applicants routinely use the selection procedure as a signal of organizational attributes (Turban, 2001; Rynes et al., 1991): the way an organization conducts its hiring process communicates information about its culture, its respect for individuals, and its trustworthiness as an employer (Gilliland, 1993; Bauer et al., 2001; Chapman et al., 2005). What turns these process cues into a value judgment is their relational meaning. In the relational, group-value model of procedural justice, fair procedures matter because they carry a relational message: they communicate to the individual their standing, respect, and value in the eyes of the party enacting them (Lind and Tyler, 1988; Tyler and Lind, 1992). Read this way, how the AI process treats an applicant tells them how much the organization values them—and it is this sense of being valued, or not, that raises or lowers the worth of joining. Organizational attractiveness (OA)—the degree to which an individual perceives an organization as a desirable place to work (Highhouse et al., 2003)—has been consistently linked to applicants’ process-based inferences in the selection literature (Chapman et al., 2005; Hausknecht et al., 2004). When applicants perceive a selection process as fair and well-designed, they are more likely to view the organization favorably and express stronger intentions to accept job offers (Bauer et al., 2001; Gilliland, 1993; Uggerslev et al., 2012). In the AI video interview context, the value process takes on particular significance because the evaluator is not a human but an algorithm. The absence of interpersonal interaction removes many of the traditional signals—warmth, empathy, responsiveness—that candidates use to evaluate employers (Langer et al., 2019; Wesche and Sonderegger, 2021). In their place, students must rely on process-level cues: does this system appear to operate fairly? Is it transparent about what it evaluates and how? Research on applicant reactions to AI-based selection confirms that these process perceptions are central to how candidates evaluate organizations that deploy such technology (Ochmann et al., 2024; Köchling et al., 2023; Acikgoz et al., 2020). We consider two process perceptions that, from a value standpoint, should most directly shape organizational attractiveness: procedural justice and algorithmic transparency.

Procedural justice refers to the perceived fairness of the procedures used to make decisions (Leventhal, 1980; Thibaut and Walker, 1975). In the selection context, procedural justice captures whether applicants perceive the hiring process as consistent, bias-free, and based on relevant criteria, and whether they are given adequate opportunity to demonstrate their abilities (Gilliland, 1993; Bauer et al., 2001). Procedural justice has long been recognized as one of the strongest predictors of applicant reactions to selection processes (Hausknecht et al., 2004; McCarthy et al., 2017): applicants who perceive the selection procedure as fair consistently report more positive attitudes toward the hiring organization (Bauer et al., 2001; Chapman et al., 2005). In AI-based selection specifically, concerns about procedural fairness are heightened. Studies show that applicants tend to perceive AI interviews as less fair than human-led formats (Acikgoz et al., 2020; Langer et al., 2020), in part because AI systems offer limited opportunity for two-way communication, provide no chance for applicants to explain or contextualize their responses, and lack the perceived empathy associated with human interviewers (Wesche and Sonderegger, 2021; Mirowska and Mesnet, 2022). When applicants perceive that the AI selection process violates procedural fairness norms—for example, by restricting their ability to perform or by appearing inconsistent—they form negative impressions of the organization as an employer (Gilliland, 1993; Köchling et al., 2023). From an SEVT value standpoint, procedural justice shapes the value process by signaling whether the organization respects and values its candidates—a relational message that, in the group-value model, conveys the applicant’s standing and worth in the organization’s eyes (Lind and Tyler, 1988; Tyler and Lind, 1992): a fair process communicates that the employer is meritocratic and trustworthy (Bauer et al., 2006; Rynes et al., 1991), thereby increasing the perceived value of joining that organization. We therefore hypothesize:

H5. Higher procedural justice is positively associated with organizational attractiveness.

Algorithmic transparency refers to the degree to which the workings, logic, and evaluation criteria of an AI system are made visible and understandable to the individuals being evaluated (Ochmann et al., 2024; Suen and Hung, 2023). It encompasses whether applicants are informed about what data the AI collects, how their responses are analyzed, and on what basis assessments are generated (Langer et al., 2021; Suen and Hung, 2023). Algorithmic transparency is conceptually related to, but distinct from, procedural justice. While procedural justice concerns whether the overall process is perceived as fair, transparency concerns whether the specific mechanisms of AI evaluation are disclosed and comprehensible (Ochmann et al., 2024). One can perceive a process as broadly fair yet still have no understanding of how the algorithm actually arrives at its assessment—and it is this opacity that generates a distinct source of discomfort for applicants (Langer et al., 2019; Lavanchy et al., 2023). In traditional face-to-face interviews, candidates can infer evaluation criteria from the questions asked and the interviewer’s reactions; in AI video interviews, these inferential cues are absent, making transparency a particularly salient concern (Nørskov et al., 2020; Langer et al., 2018). Emerging research on applicant reactions to AI selection confirms the importance of transparency: applicants who receive clear information about how the AI system works report more positive evaluations of the selection process and the organization (Langer et al., 2021; Suen and Hung, 2023), while those who perceive the system as opaque express lower organizational attractiveness and reduced willingness to apply (Wesche and Sonderegger, 2021; Acikgoz et al., 2020). Ochmann et al. (2024) further found that a substantial majority of applicants demand transparency in how they are evaluated by AI, suggesting that transparency is not a peripheral concern but a central expectation in algorithmic hiring. From an SEVT value standpoint, algorithmic transparency shapes the value process by reducing the uncertainty associated with being evaluated by a non-human agent: when organizations disclose what the AI evaluates and how assessments are generated, this openness signals respect for candidates (Gilliland, 1993; Bauer et al., 2006) and reduces the uncertainty inherent in being evaluated by a non-human agent (Langer et al., 2018; Walker et al., 2013), thereby increasing the value, for the student, of pursuing an employer willing to be this open with them—an act that itself signals they are worth informing (Chapman et al., 2005). We therefore hypothesize:

H6. Higher algorithmic transparency is positively associated with organizational attractiveness.

### 2.4 Boundary conditions

The preceding hypotheses specify which factors should predict perceived employability and organizational attractiveness, but they do not address whether these relationships hold equally for all students. SEVT emphasizes that expectancy and value beliefs are situated—shaped by individual histories, social contexts, and the characteristics that students bring to a given situation (Eccles and Wigfield, 2020). Two individual differences are particularly relevant to AI video interviews: social comparison orientation and AI usage experience. Social comparison orientation concerns how individuals evaluate themselves in relation to others (Gibbons and Buunk, 1999)—a process of self-evaluating one’s own abilities against relevant, comparable others (Festinger, 1954; Goethals and Darley, 1977) and therefore most directly relevant to the expectancy pathway, where students are assessing their own capacity. AI usage experience concerns the extent of prior experience with AI technologies (Venkatesh et al., 2003), experience that builds the elaborated schemas through which external process cues are interpreted (Alba and Hutchinson, 1987) and is therefore most directly relevant to the value pathway, where students are evaluating the organization based on how the AI process is conducted.

Social comparison orientation (SCO) refers to individuals’ dispositional tendency to compare their abilities, opinions, and outcomes with those of others (Gibbons and Buunk, 1999; Buunk and Gibbons, 2007). Individuals high in SCO habitually attend to information about how others perform and use this information to evaluate themselves (Buunk et al., 2003; Gerber et al., 2018). In educational and career contexts, social comparison has been shown to influence self-evaluation, motivation, and academic self-concept (Marsh and Parker, 1984; Dijkstra et al., 2008). The expectancy process, as conceptualized in SEVT, concerns students’ confidence in their own capacity—a judgment that is inherently self-referential. However, for students high in SCO, this self-assessment is not formed in isolation; it is continually calibrated against perceived peer performance (Gibbons and Buunk, 1999). In the AI interview context, where objective performance feedback is absent (Langer et al., 2018; Nørskov et al., 2020), students high in SCO should be particularly inclined to compare themselves with peers who have also undergone or are preparing for AI interviews. This comparison process introduces an external reference point into what is otherwise an internal self-assessment, which may alter how strongly individual factors translate into employability confidence. When students high in SCO possess strong individual factors—high metacognition, relevant AI knowledge, or intrinsic cognitive motivation—awareness that they are well-equipped relative to peers may amplify the positive association between these resources and perceived employability (Collins, 1996; Huguet et al., 2009). Conversely, students low in SCO should rely more on their own internal standards, making the relationship between individual factors and perceived employability less susceptible to social reference points. Two questions follow: what, precisely, do applicants compare, and why should comparison bear on some expectancy resources but not others. Because AI video interviews return no performance feedback (Langer et al., 2018; Nørskov et al., 2020), applicants cannot compare outcomes; what remains available for comparison is preparedness—how much one knows about what the system evaluates, and how ready one feels relative to peers facing the same process. Social comparison, however, requires an attribute that is not only relevant to the judgment at hand but also observable on a shared referent (Festinger, 1954; Goethals and Darley, 1977; Wood, 1989). AI literacy satisfies both conditions: it is explicit, declarative knowledge about a widely discussed technology, and students routinely observe how much their peers know about AI systems and how they are preparing, which supplies an external benchmark against which their own knowledge can be placed. Metacognition, need for cognition, and technology anxiety do not: the regulation of one’s own thinking, the depth of one’s cognitive engagement, and felt apprehension are internal states to which peers have no direct access, leaving no salient referent for a comparison disposition to act upon. We therefore hypothesize:

H7a. Social comparison orientation positively moderates the association between AI literacy and perceived employability, such that the association is stronger at higher levels of social comparison orientation.

AI usage experience refers to the extent of individuals’ prior use of and exposure to AI technologies in everyday and academic settings. Alba and Hutchinson (1987) separate familiarity, meaning accumulated experience with a class of stimuli, from expertise, the ability to perform related tasks successfully. AI literacy is the expertise term in our model and AI usage experience the exposure term. Literacy answers *how well can I perform in this interview; usage experience answers is this a well-designed interview. The second governs h*ow process cues are read, which makes usage experience a boundary condition on the value pathway rather than a further competence dimension within the expectancy pathway. Prior research on technology adoption suggests that familiarity with a technology shapes how individuals interpret and respond to new encounters with that technology (Venkatesh et al., 2003; Kim and Forsythe, 2008). In the context of applicant reactions, experience with technology-mediated selection has been shown to influence fairness perceptions and organizational evaluations (Langer et al., 2020; McCarthy et al., 2017). The value process, as conceptualized in SEVT, concerns students’ evaluation of the hiring organization based on how the AI selection process is conducted. We argue that students with more AI usage experience are better equipped to detect, interpret, and differentiate process cues such as fairness and transparency. Because they have interacted with AI systems across multiple contexts, they have developed richer evaluative schemas for what constitutes a well-designed versus poorly designed AI process. Exposure, distinct from competence, builds the elaborated knowledge structures through which cues are interpreted (Alba and Hutchinson, 1987; Venkatesh et al., 2003; Parasuraman, 2000). When these students encounter an AI video interview, they can more readily distinguish whether the process meets or violates their expectations, which makes process perceptions more diagnostic of organizational quality and therefore more influential in shaping organizational attractiveness. Students with little AI usage experience, by contrast, lack a comparative frame of reference and may have difficulty distinguishing between procedural features that reflect organizational values and those that are simply inherent to the AI format itself (Langer et al., 2019), attenuating the influence of process perceptions on their organizational evaluations. AI usage experience should not condition the expectancy pathway. Expectancy beliefs concern whether the applicant can perform successfully, and what raises or lowers that judgment is competence: knowing what the system assesses and how to present oneself accordingly. The model already captures this in the direct association between AI literacy and perceived employability. Exposure supplies a comparative frame for evaluating a process, not a resource for performing within it. To the extent that repeated exposure does build performance-relevant competence, that variance is captured by AI literacy itself, leaving no distinct role for usage experience as a boundary condition on the expectancy pathway. We therefore hypothesize:

H7b. AI usage experience positively moderates the relationships in the value pathway, such that the associations of process perceptions with organizational attractiveness are stronger for students who use AI tools more frequently.

We summarize all hypotheses in Figure 1.

**Figure 1:**
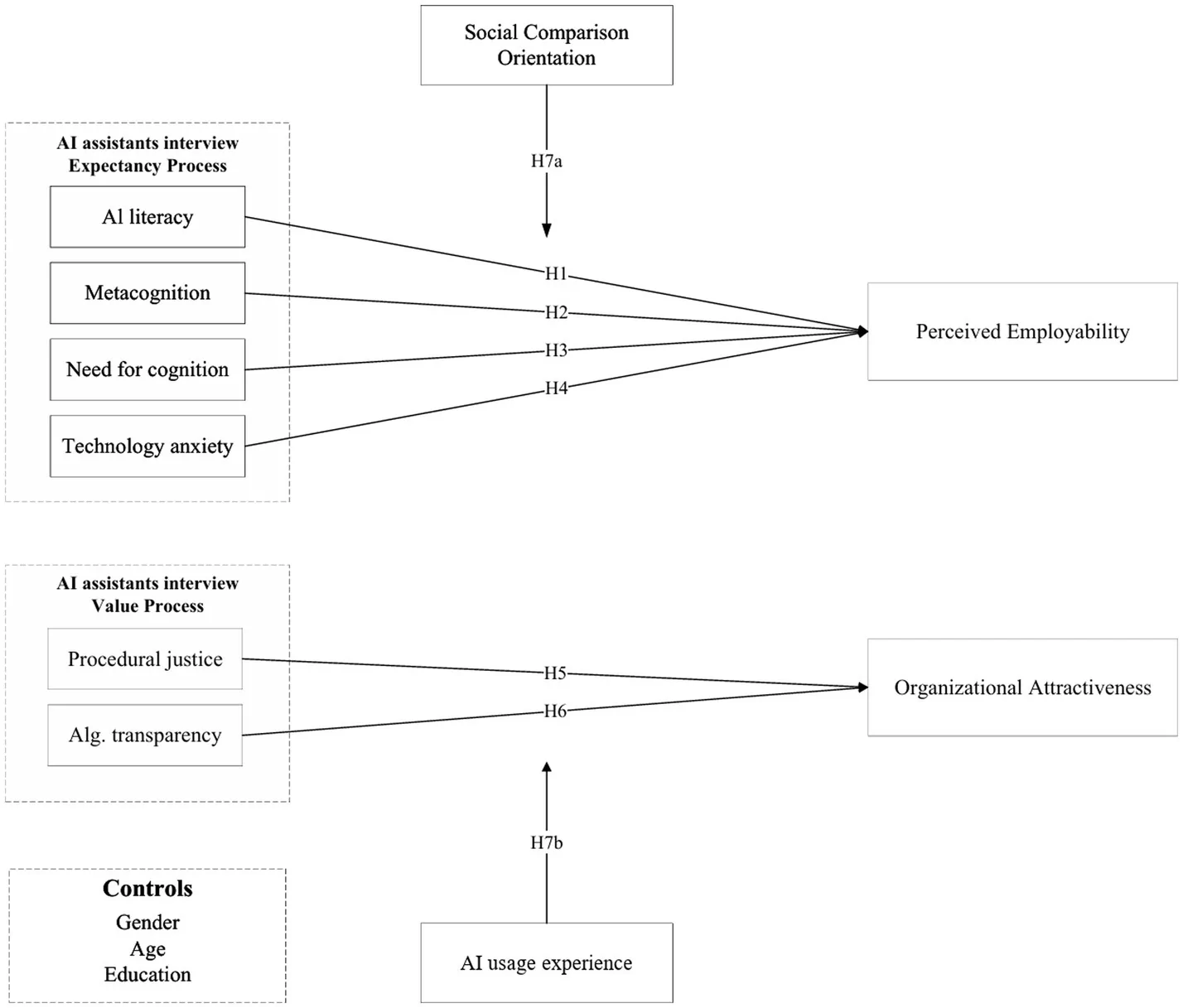
Research model and hypotheses.

## 3 Method

### 3.1 Sample and data collection

Data were collected from university students in China using a scenario-based online survey. University students were selected as the target population because they are increasingly likely to encounter AI video interviews as part of the graduate recruitment process (Woods et al., 2020), they represent the career stage at which AI-mediated selection first becomes relevant (Langer et al., 2019), and they provide valid self-report data on job-seeking attitudes and career perceptions (Chapman et al., 2005). The scenario-based approach was adopted because not all participants had direct experience with AI video interviews, and scenario designs are widely used in recruitment research to study applicant reactions to selection procedures (Lievens and Highhouse, 2003; Walker et al., 2013).

Data were collected through the Credamo platform, a widely used Chinese online survey platform for academic research, from December 2025 to February 2026. Before responding to the main questionnaire, participants were presented with a recruitment scenario. They were asked to imagine that they had passed an initial resume screening and were now proceeding to an AI video interview. The scenario instructed them to envision interacting with an AI virtual interviewer that would pose structured questions while simultaneously analyzing their verbal expressions, facial expressions, and response content to generate an overall assessment. A visual illustration of the AI interview interface was provided to enhance the vividness of the scenario. Participants then completed the survey based on how they would feel and what they would think in such a situation. This scenario design follows established practices in applicant reactions research (Lievens and Highhouse, 2003; Walker et al., 2013) and ensures that all respondents evaluated the same AI interview context. A total of 606 questionnaires were collected. Following data screening, 86 responses were excluded due to incomplete answers, excessively short completion times (<3 min), failed attention check items, and straight-lining response patterns, yielding a final sample of 520 valid responses (effective rate: 85.8%). Sample size adequacy was verified using G*Power 3.1 (Faul et al., 2009): with seven predictors, a medium effect size (*f*^2^ = 0.15), *α* = 0.05, and statistical power = 0.95, the minimum required sample was 153, well below our sample of 520. Table 1 presents the demographic profile. Among the 520 participants, 376 (72.3%) were female and 144 (27.7%) were male. The mean age was 23.23 years (SD = 2.77), with the majority aged between 20 and 25. In terms of education, 276 (53.1%) held or were pursuing a bachelor’s degree and 210 (40.4%) held or were pursuing a master’s degree. Regarding AI interview experience, 237 participants (45.6%) reported no prior experience with AI-based interviews, 202 (38.8%) had experienced one AI interview, and 81 (15.6%) had experienced two or more.

**Table 1:** Demographic characteristics (*N* = 520).

Variable	Category	Frequency	%
Gender	Male	144	27.7
	Female	376	72.3
Age (years)	18–20	89	17.1
	21–23	246	47.3
	24–26	129	24.8
	≥27	56	10.8
	Mean (SD)	23.23 (2.77)	
Education	Junior college	28	5.4
	Bachelor’s	276	53.1
	Master’s	210	40.4
	Doctoral	6	1.2
AI interview exp.	None	237	45.6
	1 time	202	38.8
	2–3 times	66	12.7
	≥4 times	15	2.9
AI tool usage	Never	5	1.0
	Rarely	30	5.8
	Sometimes	127	24.4
	Often	130	25.0
	Very frequently	228	43.8

### 3.2 Measures

All constructs were measured using established scales adapted from prior literature and translated into Chinese via back-translation (Brislin, 1970). All items were rated on seven-point Likert scales (1 = strongly disagree, 7 = strongly agree). In the expectancy pathway, AI Literacy was measured using five of the twelve items of Wang et al. (2023), capturing AI awareness, use, evaluation, and selection; the scale’s ethical-use items are not part of the composite. Metacognition was measured using five items adapted from Turban et al. (2009) and Pintrich et al. (1991), assessing job search-related goal-setting, planning, monitoring, and self-reflection; one item (MET4) was removed due to a low outer loading (0.441). Need for Cognition was measured using six items from the NCS-6 scale (Lins de Holanda Coelho et al., 2020; Cacioppo et al., 1984). Technology Anxiety was measured using seven items adapted from Meuter et al. (2003). The scale contains both anxiety-worded and confidence-worded items; the three confidence-worded items (TA1, TA5, TA8) were reverse-scored so that higher values indicate greater anxiety. Two items (TA6, TA7) were removed for low outer loadings (0.410 and 0.445). In the value pathway, Procedural Justice was measured using three items adapted from Muylle and Standaert (2016), assessing perceived consistency, non-discrimination, and respect in the AI interview process. Algorithmic Transparency was measured using a four-item semantic differential scale adapted from Martin et al. (2017) and employed by Ochmann et al. (2024), capturing the perceived clarity and comprehensibility of the AI system’s decision-making process.

Perceived Employability was measured with the 11 items retained by Rothwell and Arnold (2007), adapted for a student sample. Those items were written for employed professionals, so the referent was changed before administration; no item was added or dropped. Six of the eleven named the respondent’s current organization and two referred to their current job. In these, the organization became the target company and the current job became the skills and experience the respondent has acquired: “Even if there was downsizing in this organisation, I am confident that I would be retained” became “Even if the target company were to downsize, I am confident I would be retained.” The remaining three items already addressed the external labour market and kept their referent. The adaptation was carried out by the author team, and the Chinese wording was then produced by back-translation (Brislin, 1970). The adapted items were not submitted to a separate content-validation panel or pretest; the evidence for the measure is its performance in this sample, which met the criteria applied to every construct here (*α* = 0.922, CR = 0.934, AVE = 0.566, with discriminant validity by the Fornell–Larcker and HTMT criteria; Tables 2–4). Supplementary Material S1 gives every item as administered, in Chinese with an English back-translation and a note on the change made to each. Supplementary Material S2 states the coding rules applied before estimation: the reverse-scored items, the coding of the background and control variables, and the item purification decisions.

**Table 2:** Measurement model: factor loadings, reliability, and convergent validity.

Construct/item	Loading	*α*	CR (ρc)	AVE	ρA	FC VIF
AI Literacy (Wang et al., 2023)		0.725	0.819	0.476	0.733	1.472
I can identify the AI technology employed.	0.722					
I can skillfully use AI applications or products to help me with my daily work.	0.732					
I can evaluate the capabilities and limitations of an AI application or product after using it for a while.	0.627					
I can choose a suitable solution from the several an AI provides.	0.654					
I can choose the most appropriate AI application or product from a variety for a particular task.	0.710					
Metacognition (Turban et al., 2009; Pintrich et al., 1991)		0.798	0.859	0.553	0.834	1.567
Set personal goals to guide job search activities.	0.840					
Developed a coherent plan to guide my job search.	0.833					
Monitored my progress toward finding a job.	0.720					
Thought about how best to present myself to potential employers.	0.672					
Analyzed interview performance to improve subsequent performance.	0.630					
Need for Cognition (Cacioppo et al., 1984)		0.916	0.935	0.706	0.926	1.579
I would prefer complex to simple problems.	0.888					
I like to take on tasks that require a lot of thinking.	0.901					
Thinking is not my idea of fun. (R)	0.758					
I would rather do something simple than something that challenges my thinking. (R)	0.844					
I really enjoy finding new solutions to problems.	0.795					
I would prefer an intellectually demanding, difficult and important task to one that is less important but requires little thought.	0.845					
Technology Anxiety (adapted from Meuter et al., 2003)		0.852	0.887	0.529	0.872	2.334
I am confident I can master the skills required for an AI interview. (R)	0.801					
I find it difficult to understand how the AI interview system works.	0.720					
I feel nervous at the thought of taking an AI interview.	0.638					
Facing an AI interview, I worry that I will make operational mistakes.	0.721					
I am sure I can understand the results and feedback of an AI interview. (R)	0.695					
I can adapt to AI interviews as a new form of recruitment. (R)	0.804					
I hesitate to take an AI interview for fear that a mistake could not be undone.	0.699					
Procedural Justice (adapted from Muylle and Standaert, 2016)		0.721	0.842	0.640	0.730	1.839
Did not discriminate but rather treated all candidates similarly.	0.824					
Applied consistent policies and decision-making procedures across all candidates.	0.762					
Treated the candidates with respect.	0.812					
Algorithmic Transparency (adapted from Martin et al., 2017)		0.914	0.939	0.795	0.916	1.984
Unclear to me/Clear to me	0.879					
Confusing/Straightforward	0.881					
Difficult to understand/Easy to understand	0.913					
Vague/Transparent	0.894					
Perceived Employability (adapted from Rothwell and Arnold, 2007)		0.922	0.934	0.566	0.926	2.324
Even if the target company were to downsize, I am confident I would be retained.	0.804					
My personal network supports my career development.	0.702					
I am aware of opportunities in the job market, including roles different from my current target.	0.793					
The skills I have acquired can be applied in other organizations and roles.	0.722					
I can improve my competitiveness in the job market through further learning.	0.620					
I have a clear understanding of the range of career opportunities suited to me.	0.739					
Among comparable job seekers, my abilities are recognised.	0.815					
I could easily find a similar job at another company.	0.781					
I could obtain a similar position at most companies.	0.820					
People with my level of skills and experience are sought after by employers.	0.803					
As long as my skills and experience match, I could handle a range of jobs.	0.643					
Organizational Attractiveness (Highhouse et al., 2003)		0.913	0.938	0.792	0.913	2.370
Unattractive/Attractive	0.875					
Bad/Good	0.900					
Unpleasant/Pleasant	0.899					
Disapprove/Approve	0.886					
Social Comparison Orientation (adapted from Gibbons and Buunk, 1999)		0.869	0.897	0.494	0.875	1.109

*N* = 520. All outer loadings significant at *p* < 0.001 based on 10,000 bootstrap resamples. Three items (MET4, TA6, TA7) were removed due to outer loadings < 0.45. (*R*) = reverse-coded item. FC VIF = full collinearity VIF (Kock, 2015); all values <3.3. SCO item loadings are omitted for space. AIL AVE (0.476) falls marginally below the 0.50 benchmark but is acceptable given CR > 0.70 (Hair et al., 2019). Item wording is the English back-translation of the item as administered in Chinese, not the wording of the source scale. The full administered instrument, including items not retained in the reported model, is provided in the supplementary materials.

**Table 3:** Descriptive statistics and Fornell–Larcker latent-variable correlations.

	M	SD	1	2	3	4	5	6	7	8
1. AIL	5.542	0.683	0.690							
2. MC	5.610	0.832	0.400	0.744						
3. NFC	4.639	1.350	0.289	0.356	0.840					
4. TA	3.178	1.061	−0.429	−0.333	−0.479	0.727				
5. PJ	5.225	1.203	0.290	0.152	0.204	−0.499	0.800			
6. AT	4.837	1.380	0.371	0.257	0.262	−0.604	0.526	0.892		
7. PE	5.013	0.992	0.503	0.575	0.564	−0.553	0.313	0.376	0.752	
8. OA	4.612	1.348	0.321	0.300	0.316	−0.608	0.642	0.628	0.405	0.890

*N* = 520. M = mean; SD = standard deviation. Bold diagonal entries are the square roots of the AVE; off-diagonal entries are latent-variable correlations. TA is coded so that higher values indicate greater anxiety. SCO (M = 5.429, SD = 0.872) and AI usage experience (M = 3.050, SD = 1.000) are reported in text.

**Table 4:** Heterotrait–monotrait (HTMT) ratio of correlations.

	AIL	MC	NFC	TA	PJ	AT	PE	OA
AIL	—							
MC	0.517	—						
NFC	0.339	0.385	—					
TA	0.513	0.373	0.533	—				
PJ	0.391	0.196	0.247	0.616	—			
AT	0.442	0.289	0.280	0.669	0.646	—		
PE	0.607	0.647	0.607	0.605	0.383	0.407	—	
OA	0.384	0.332	0.340	0.677	0.782	0.686	0.438	—

*N* = 520. All HTMT values fall below the conservative 0.85 threshold (Henseler et al., 2015).

Organizational Attractiveness was measured using a four-item semantic differential scale (Highhouse et al., 2003), assessing respondents’ overall attitude toward the organization (unattractive/attractive, bad/good, unpleasant/pleasant, disapprove/approve). Social Comparison Orientation was measured using nine of the 11 administered items from Gibbons and Buunk (1999), adapted to the job search context. AI usage experience was measured with a single indicator recording how frequently respondents had used AI tools in the preceding week. Previous AI interview experience was recorded separately and is reported as a sample characteristic. Appendix A, (Table A5) reports a sensitivity analysis in which the two indicators are entered as moderators separately and as a two-item composite; the moderation in the value pathway is carried by AI usage experience alone. Control variables included gender, age, and education level (Jackson and Tomlinson, 2020; Langer et al., 2019). Table 2 reports the full measurement model results.

### 3.3 Data analysis

Partial least squares structural equation modeling (PLS-SEM) was employed to test the proposed model, using the seminar package (version 2.3.7) in R (Ray et al., 2022). PLS-SEM was chosen over covariance-based SEM (CB-SEM) for several reasons. First, the analytical objective is to explain variance in the endogenous constructs (perceived employability and organizational attractiveness) and to assess whether the model holds out of sample, both of which align with the strengths of PLS-SEM (Hair et al., 2019, 2021); out-of-sample performance is examined with PLSpredict and reported in Section 4.2. Second, the data violated the assumption of multivariate normality (Shapiro–Wilk tests, all *p* < 0.001), making PLS-SEM’s distribution-free estimation more appropriate. Third, the model involves a relatively complex structure with multiple independent variables, two dependent variables, control variables, and moderation effects that are best analyzed using the two-stage approach available in PLS-SEM (Becker et al., 2023). All constructs were modeled as Mode A composites, the standard specification in PLS-SEM (Hair et al., 2021).

Following the two-step analytical procedure recommended by Anderson and Gerbing (1988), the measurement model was assessed first to ensure adequate reliability and validity, followed by the structural model. Model estimation was accompanied by bootstrapping with 10,000 resamples to obtain standard errors, *t*-statistics, and bias-corrected 95% confidence intervals for all path coefficients (Hair et al., 2021). Items with outer loadings below 0.45 were removed following Hair et al. (2019), resulting in the deletion of three items: MET4 (loading = 0.441), TA6 (loading = 0.410), and TA7 (loading = 0.445). Moderation was tested using the two-stage approach (Becker et al., 2023; Hair et al., 2021), in which latent variable scores from the base model are used to create interaction terms that are then entered as predictors in a second estimation. H7a predicts that social comparison orientation moderates the association between AI literacy and perceived employability, and H7b predicts that AI usage experience moderates the two value-pathway associations. To avoid selective reporting, all twelve interactions of the two moderators with the six antecedents were estimated; the three hypothesized interactions are reported in Table 5 and the remaining nine, which were not hypothesized, are reported as exploratory in Appendix A, (Table A2).

**Table 5:** Within-pathway moderation tests (H7a, H7b).

Interaction	DV	*β*	SE	*t*	95% CI	*f* ^2^	Decision
Panel A: Social comparison orientation × expectancy pathway (H7a: AI literacy; remaining tests exploratory)							
SCO × AIL → PE	PE	0.076	0.028	2.75	[0.013, 0.122]	0.020	Supported
SCO × MC → PE	PE	−0.001	0.034	−0.03	[−0.070, 0.068]	<0.001	Exploratory
SCO × NFC → PE	PE	0.003	0.032	0.09	[−0.065, 0.059]	<0.001	Exploratory
SCO × TA → PE	PE	0.010	0.037	0.26	[−0.057, 0.088]	<0.001	Exploratory
Panel B: AI usage experience × value pathway (H7b)							
AI Use × PJ → OA	OA	0.085	0.027	3.11	[0.030, 0.138]	0.019	Supported
AI Use × AT → OA	OA	0.076	0.028	2.69	[0.020, 0.132]	0.015	Supported

*N* = 520. Two-stage moderation (Becker et al., 2023; Hair et al., 2021) with 10,000 bootstrap resamples. H7a predicts the SCO × AI literacy interaction only; the three remaining SCO interactions in Panel A were examined on an exploratory basis and are labelled accordingly. H7b predicts both interactions in Panel B. The nine interactions not shown here are reported in Appendix A, (Table A2).

### 3.4 Common method bias

Because all data were collected through a single self-report questionnaire at one point in time, common method bias (CMB) is a potential concern. Both procedural and statistical remedies were employed (Podsakoff et al., 2003). Procedurally, respondents were assured of anonymity and confidentiality, predictor and criterion variables were placed in separate sections of the questionnaire, different scale formats were used across constructs (seven-point Likert and semantic differential), and item order was randomized within sections. Statistically, Harman’s single-factor test showed that the first unrotated factor accounted for 25.7% of the total variance, well below the 50% threshold. Full collinearity VIF values ranged from 1.109 to 2.370, all below the 3.3 threshold (Kock, 2015). A single-factor confirmatory model fit the data poorly (CFI = 0.45, RMSEA = 0.10, SRMR = 0.12). An unmeasured latent method factor accounted for 11.0% of the variance on average, versus 43.1% for the substantive constructs. CMV-adjusting the construct correlations via Lindell and Whitney’s (2001) marker-variable procedure left every hypothesized relationship significant. These results suggest that CMB is unlikely to be a serious threat to the validity of the findings.

## 4 Results

### 4.1 Measurement model

The measurement model was assessed in terms of indicator reliability, internal consistency reliability, convergent validity, and discriminant validity, following the guidelines of Hair et al. (2021). Indicator reliability was evaluated through outer loadings. After removing three items with loadings below 0.45, all remaining indicator loadings ranged from 0.620 (PE5) to 0.913 (ALT3), with the majority exceeding 0.70 (Table 2). Internal consistency reliability was evaluated using Cronbach’s alpha (*α*) and composite reliability (ρc). Cronbach’s alpha values ranged from 0.721 (PJ) to 0.922 (PE), and composite reliability values ranged from 0.819 (AIL) to 0.939 (OA), all exceeding the 0.70 threshold (Hair et al., 2021). Convergent validity was assessed through the average variance extracted (AVE). Most constructs exhibited AVE values above 0.50 (Fornell and Larcker, 1981): MC (0.553), NFC (0.706), TA (0.529), PJ (0.640), AT (0.795), PE (0.566), and OA (0.792). The AVE of AI literacy (0.476) fell marginally below 0.50; however, its composite reliability (0.819) was well above 0.70 and all indicator loadings exceeded 0.60, which is considered acceptable (Hair et al., 2019).

Discriminant validity was assessed using two complementary approaches. First, the Fornell–Larcker criterion was examined: the square root of each construct’s AVE (shown in bold on the diagonal of Table 3) exceeded its correlations with all other constructs (Fornell and Larcker, 1981). Second, the heterotrait-monotrait (HTMT) ratio was computed (Table 4). All HTMT values fell below the conservative 0.85 threshold (Henseler et al., 2015), with the highest value being 0.782 for the PJ–OA pair. This value is consistent with theoretical expectations, as procedural justice is among the strongest known predictors of organizational attractiveness in selection contexts (Bauer et al., 2001; Hausknecht et al., 2004). Together, these results support the empirical distinctness of the constructs.

### 4.2 Structural model: direct paths

Before assessing the structural model, variance inflation factor (VIF) values were examined for all predictor constructs. All inner VIF values were below 3.0, indicating that multicollinearity was not a concern (Hair et al., 2021). The structural model was evaluated in terms of path coefficients (*β*), statistical significance (*t*-values and 95% bias-corrected bootstrap confidence intervals), explained variance (*R*^2^), and effect sizes (*f*^2^). Path coefficients are reported using conventional structural-equation terminology; because the design is cross-sectional, all coefficients should be interpreted as associations rather than causal effects.

Table 6 reports the six direct paths. All paths were significant at *p* < 0.001. Within the expectancy pathway, the model explained 58.1% of the variance in perceived employability (*R*^2^ = 0.581), indicating substantial explanatory power (Cohen, 1988). The largest coefficient was for metacognition (*β* = 0.322, *t* = 7.27, *f*^2^ = 0.186), followed by need for cognition (*β* = 0.258, *t* = 5.61, *f*^2^ = 0.108), technology anxiety (*β* = −0.217, *t* = −4.58, *f*^2^ = 0.074), and AI literacy (*β* = 0.190, *t* = 4.48, *f*^2^ = 0.062). According to Cohen’s (1988) benchmarks, the effect size for metacognition was medium, that for NFC small-to-medium, and those for technology anxiety and AI literacy small. H1 through H4 were therefore supported. Pairwise differences between the four coefficients were tested against their bootstrap distributions (10,000 resamples). None was significant; the largest, metacognition versus AI literacy, gave Δ = 0.131, 95% CI [−0.012, 0.277], *p* = 0.071. The ordering above is therefore descriptive. A relative weights analysis (Johnson, 2000; Budescu, 1993), reported in Appendix A, (Table A3), decomposes the explained variance among the four predictors and supports the same reading: the four contributions do not differ significantly from one another.

**Table 6:** Structural model results: direct paths (H1–H6).

Hypothesis	Path	*β*	SE	*t*	95% CI	*f* ^2^	Decision
Individual resource pathway (*R*^2^ = 0.581)							
H1	AIL → PE	0.190	0.042	4.48	[0.108, 0.275]	0.062	Supported
H2	MC → PE	0.322	0.044	7.27	[0.236, 0.409]	0.186	Supported
H3	NFC → PE	0.258	0.046	5.61	[0.170, 0.349]	0.108	Supported
H4	TA → PE	−0.217	0.047	−4.58	[−0.311, −0.125]	0.074	Supported
Process perception pathway (*R*^2^ = 0.541)							
H5	PJ → OA	0.415	0.042	9.96	[0.333, 0.497]	0.261	Supported
H6	AT → OA	0.393	0.043	9.10	[0.305, 0.476]	0.242	Supported

*N* = 520; 10,000 bootstrap resamples; all *p* < 0.001. Controls: Gender → PE (*β* = 0.087, *p* < 0.01), Gender → OA (*β* = 0.067, *p* < 0.05), Age → PE (*β* = 0.071, *p* < 0.05), Age → OA (*β* = 0.064, *p* = 0.082), Education → PE (*β* = −0.081, *p* < 0.05), Education → OA (*β* = −0.088, *p* = 0.052).

Within the value pathway, the model explained 54.1% of the variance in organizational attractiveness (*R*^2^ = 0.541). Procedural justice (*β* = 0.415, *t* = 9.96, *f*^2^ = 0.261) and algorithmic transparency (*β* = 0.393, *t* = 9.10, *f*^2^ = 0.242) were both associated with organizational attractiveness, and both effect sizes were medium-to-large. The difference between them was not significant (Δ = 0.024, 95% CI [−0.131, 0.182], *p* = 0.754). H5 and H6 were supported. Figure 2 displays the six path coefficients as a forest plot with 95% bootstrap confidence intervals.

**Figure 2:**
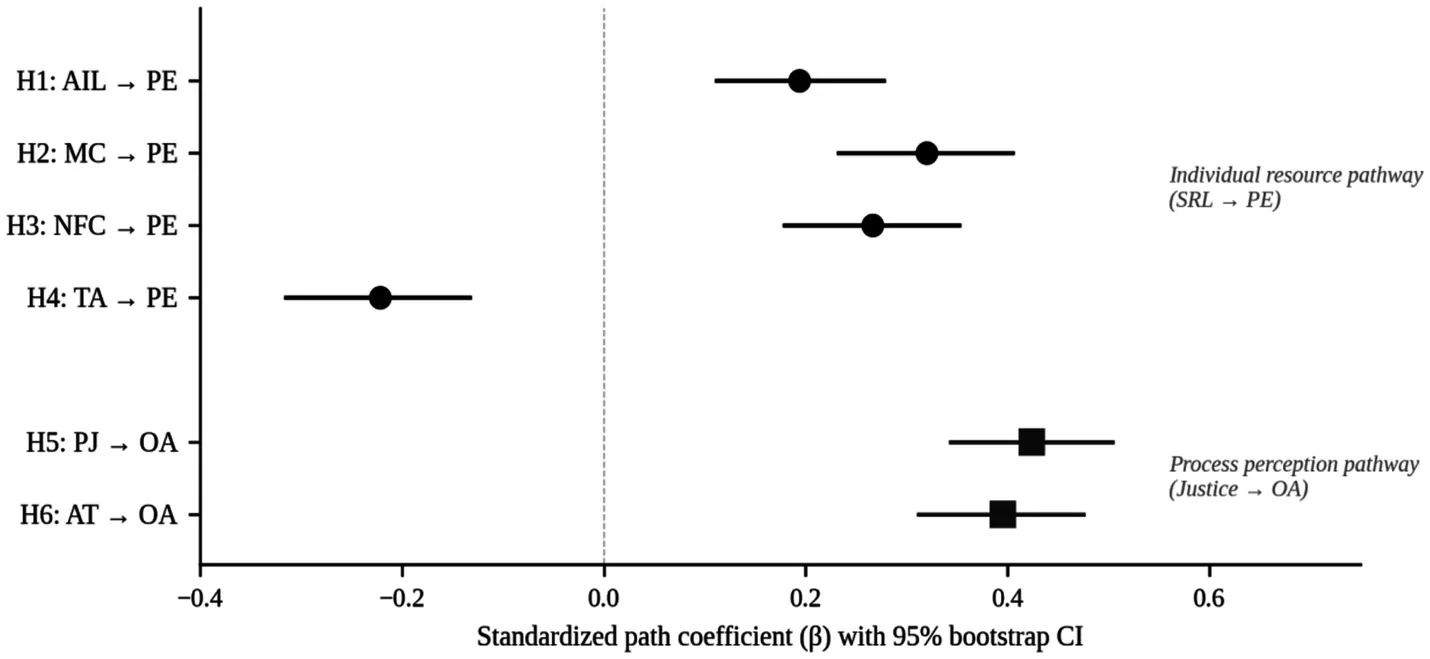
Forest plot of direct paths (H1–H6) with 95% bootstrap confidence intervals.

Among the control variables, gender was positively associated with both perceived employability (*β* = 0.087, *t* = 3.15, *p* < 0.01) and organizational attractiveness (*β* = 0.067, *t* = 2.18, *p* < 0.05), indicating that male students reported slightly higher scores on both outcomes. Age was positively associated with perceived employability (*β* = 0.071, *t* = 2.30, *p* < 0.05) and marginally with organizational attractiveness (*β* = 0.064, *t* = 1.75, *p* = 0.082). Education was negatively associated with perceived employability (*β* = −0.081, *t* = −2.33, *p* < 0.05) and marginally with organizational attractiveness (*β* = −0.088, *t* = −1.95, *p* = 0.052).

A model in which all six antecedents predicted both outcomes is reported in Appendix A, (Table A1). Neither procedural justice (*β* = 0.038, *t* = 0.95) nor algorithmic transparency (*β* = −0.002, *t* = −0.05) was associated with perceived employability, and adding them raised *R*^2^ by less than 0.001. Organizational attractiveness drew on two of the four individual factors: metacognition (*β* = 0.108, *t* = 2.83) and technology anxiety (*β* = −0.243, *t* = −5.33) were associated with it, whereas AI literacy (*β* = −0.045) and need for cognition (*β* = 0.023) were not. All six hypothesized paths remained significant, and the two process perceptions remained the strongest predictors of organizational attractiveness. Section 5.1 returns to the technology anxiety result. Out-of-sample predictive relevance was assessed with PLSpredict (ten-fold cross-validation, ten repetitions). Q^2^predict exceeded zero for all fifteen indicators (0.203 to 0.451). Relative to a linear-regression benchmark, prediction error was lower for ten of the eleven perceived employability indicators and for two of the four organizational attractiveness indicators, placing the model’s predictive advantage in the expectancy pathway.

### 4.3 Moderation tests

Table 5 reports the moderation tests for the three hypothesized interactions. Consistent with H7b, AI usage experience moderated both value-pathway associations: AI Use × PJ → OA (*β* = 0.085, *t* = 3.11, 95% CI [0.030, 0.138], *f*^2^ = 0.019) and AI Use × AT → OA (*β* = 0.076, *t* = 2.69, 95% CI [0.020, 0.132], *f*^2^ = 0.015). H7b was supported: students who used AI tools more frequently showed stronger associations between process perceptions and organizational attractiveness, consistent with the reasoning that experienced users are more discerning evaluators of AI process quality.

H7a predicted that social comparison orientation moderates the association between AI literacy and perceived employability. This interaction was significant (*β* = 0.076, *t* = 2.75, 95% CI [0.013, 0.122], *f*^2^ = 0.020), and H7a was therefore supported. The three remaining social comparison interactions were examined on an exploratory basis rather than hypothesized, and none was significant (all |*t*| < 0.30). This selective pattern suggests that AI literacy—as an explicit, socially visible form of knowledge—is more amenable to social-comparison amplification than metacognitive regulation, cognitive engagement style, or affective responses to technology, which are more internal and less readily compared across peers.

Taken together, the moderation results indicate a clearer boundary condition on the value pathway than on the expectancy pathway: AI usage experience moderated both process-perception associations, whereas social comparison orientation moderated only the AI literacy association. Of the nine exploratory interactions, one was significant (AI usage experience × technology anxiety → perceived employability, *β* = 0.091, *t* = 2.94), which is what nine tests at *α* = 0.05 would be expected to produce by chance. All moderation effect sizes were small (*f*^2^ = 0.015 to 0.022), and the moderation results should therefore be read as qualifying rather than reshaping the direct associations. Figure 3 shows the simple slopes for the three hypothesized interactions at Low (−1 SD), Mean, and High (+1 SD) levels of the moderators.

**Figure 3:**
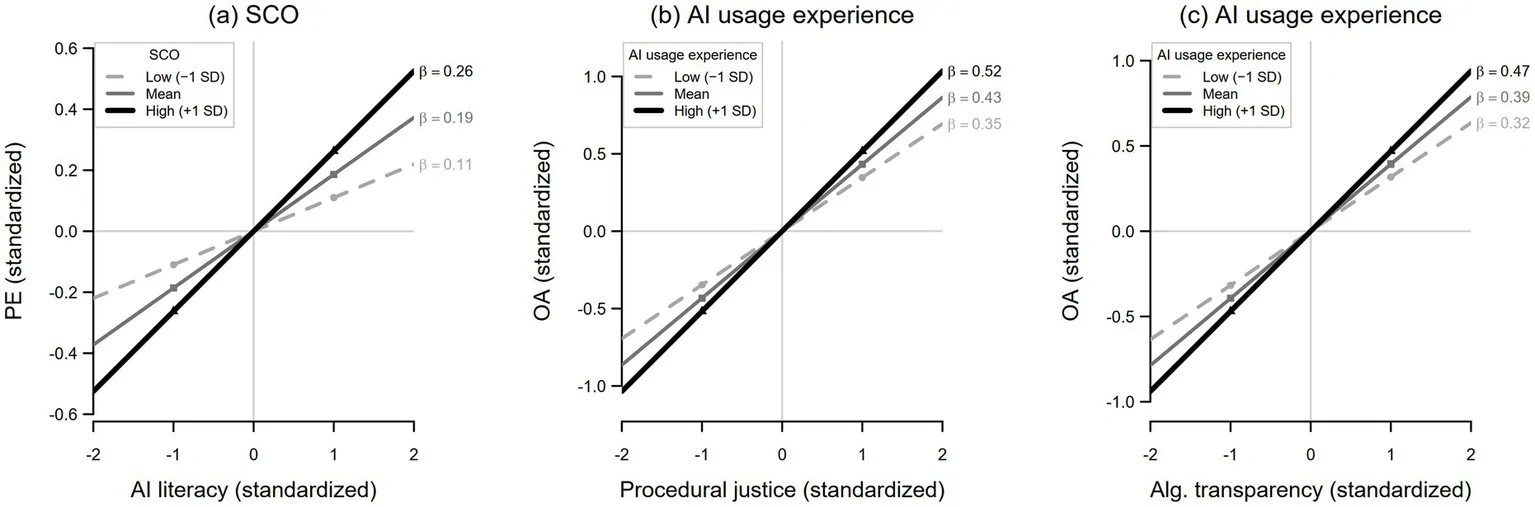
Simple slopes for the three hypothesized interactions at Low (−1 SD), Mean, and High (+1 SD) levels of the moderator. **(a)** SCO × AIL → PE; **(b)** AI use × PJ → OA; **(c)** AI use × AT → OA.

## 5 General discussion

This study set out to address a gap in the existing literature: employability confidence and organizational attractiveness have been studied as separate outcomes, leaving unclear which factors matter most for each and where universities should focus their limited preparation resources. Drawing on Situated Expectancy-Value Theory, we developed and tested a dual-pathway model that organizes the antecedents of successful employment into an expectancy pathway and a value pathway. Using survey data from 520 Chinese university students, all six direct hypotheses were supported, and the two career outcomes were associated with partially differentiated sets of antecedents. All four expectancy-pathway factors were independently associated with employability confidence, and their coefficients did not differ from one another. Three of the four are unrelated to AI-specific knowledge, so cognitive engagement and emotional readiness contribute alongside what students know about AI. Procedural justice and algorithmic transparency were both substantially associated with organizational attractiveness, with transparency reaching a medium-to-large effect in a literature where procedural justice has typically dominated. The moderation analyses added further nuance: AI usage experience moderated both value-pathway links (H7b supported), with associations stronger at higher levels of use. Social comparison orientation moderated the AI literacy association within the expectancy pathway (H7a supported), while the three exploratory interactions in that pathway were not significant, indicating that boundary conditions operate selectively rather than uniformly across the model. Below we discuss the theoretical contributions, practical implications, and limitations of these findings.

### 5.1 Theoretical contributions

First, this study extends SEVT to the context of AI-mediated recruitment, providing a unified theoretical framework for understanding how AI-assisted interviews are repositioned as an evaluative authority, relating to both sides of employment decisions. Prior research has examined factors related to employability confidence (Shi and Wang, 2025; Bennett and Ananthram, 2022) and organizational attractiveness (Langer et al., 2019; Ochmann et al., 2024) as separate lines of inquiry. By mapping individual factors and process perceptions onto SEVT’s expectancy and value components, our results indicate that these two outcome domains have partially differentiated antecedent structures. The existing literature has not formalized that distinction, and the supplementary model reported in Section 4.2 shows it to be asymmetric rather than absolute. The expectancy pathway and the value pathway each explained substantial variance, confirming that both evaluative processes are consequential for students’ career development and that neither can be reduced to the other. More broadly, delegating evaluation to an opaque algorithm repositions where applicants locate competence and worth: without the interpersonal exchange that traditionally scaffolds these judgments, students must appraise both their own standing and the organization’s legitimacy from the conduct of the algorithmic process itself. That the two appraisals are associated with partially differentiated antecedent patterns, rather than with one common set, suggests that responses to algorithmic authority are not a single global reaction. The differentiation is asymmetric rather than complete: neither process perception was associated with perceived employability, whereas technology anxiety and metacognition were also associated with organizational attractiveness (Appendix A, Table A1).

Second, the four expectancy-pathway factors were estimated together rather than one at a time, which prior research on responses to algorithmic selection has rarely done. With AI literacy held constant, metacognition, need for cognition, and technology anxiety each remained associated with employability confidence, and the four coefficients did not differ from one another. AI-specific knowledge is therefore one of several resources students draw on in appraising themselves under algorithmic evaluation, not the resource that subsumes the others. Competence in this setting is composite, spanning self-regulatory, motivational, and affective capacities alongside knowledge about the technology (Long and Magerko, 2020; Ng et al., 2021; World Economic Forum, 2025). This accords with Bennett and Ananthram’s (2022) account of perceived employability as formed within graduates’ metacognitive framework. Within the value pathway, algorithmic transparency was associated with organizational attractiveness at medium-to-large magnitude, which extends the applicant reactions literature (Bauer et al., 2001; Hausknecht et al., 2004) to the AI recruitment context by placing transparency alongside procedural fairness rather than behind it.

The value pathway was not closed to individual factors. In the supplementary model, technology anxiety was related to organizational attractiveness (*β* = −0.243) and metacognition weakly so (*β* = 0.108), while AI literacy and need for cognition were not. Situated expectancy-value theory accounts for the technology anxiety result. Subjective task value comprises attainment, intrinsic, and utility components together with cost, and cost includes the emotional burden a person expects to bear in pursuing an outcome (Eccles and Wigfield, 2020). An applicant who finds the evaluating technology aversive expects both to perform less well in the interview and to bear more in working for an employer that screens this way, so technology anxiety lowers expectancy and raises anticipated cost at the same time. Affect therefore reaches the value side of the model, whereas what students know about AI and how readily they engage effortful tasks were associated only with the expectancy side in this sample. The weaker metacognition association admits no equally direct reading, and we report it without one. Neither association was hypothesized, and both require direct testing.

Third, the moderation results indicate that the strength of these associations varies with individual differences in selective and interpretable ways. Both value-pathway associations were stronger among students who use AI more frequently, indicating that frequent users were more responsive to process cues rather than less: they may have developed richer evaluative schemas that make process cues more diagnostic of organizational quality. Social comparison orientation moderated only the AI literacy association within the expectancy pathway; the associations of metacognition, NFC, and technology anxiety did not vary with it. This selectivity was predicted rather than discovered: comparison requires a referent, and AI literacy is an explicit, socially visible form of knowledge that is readily compared across peers, whereas metacognitive self-regulation, cognitive engagement, and affective responses to technology are more internal processes that are less amenable to social benchmarking (Gibbons and Buunk, 1999).

### 5.2 Practical implications

AI video interviews are rapidly becoming the default first-stage screening method in graduate recruitment, yet university career preparation programs have been designed primarily around traditional interview formats and AI literacy curricula, with limited attention to the two sets of perceptions that accompany selection through algorithmic channels. In the Introduction, we identified a dual challenge facing university students: they must simultaneously build confidence in their own employability and maintain their willingness to join organizations that use algorithmic selection. We also noted that university preparation resources are finite and that existing research provides insufficient guidance on where these resources should be concentrated. Our findings speak directly to these concerns.

For universities, the results bear on how preparation resources might be allocated across the two conditions that jointly matter for successful employment. On the employability side, we asked whether universities should prioritize AI literacy or broader self-regulatory competencies. As reported above, metacognition, need for cognition, and technology anxiety each remained associated with employability confidence, and the four coefficients did not differ from one another. The results do not rank these resources. They do indicate that career preparation should not treat AI literacy as its sole investment. Whether adding metacognitive training to existing curricula raises employability confidence is a causal question this design cannot settle and one worth testing directly. The significant role of technology anxiety further suggests that emotional readiness deserves dedicated attention; low-stakes mock AI interview practice may help reduce anxiety before students face actual algorithmic evaluation. On the organizational attractiveness side, both procedural fairness and algorithmic transparency were associated with organizational attractiveness at medium-to-large magnitude, and our data do not determine which is the more consequential. Career counseling should therefore equip students not only to recognize whether an AI process treats them fairly, but also to evaluate whether the system is transparent about its evaluation criteria—a distinction that current preparation programs rarely make explicit.

For students, the moderation findings suggest that a uniform preparation strategy may not be optimal. Students high in social comparison orientation derive additional employability confidence from AI literacy when they perceive themselves as better-prepared than peers, suggesting that AI knowledge serves a social-comparative function for this subgroup. Students who use AI more often are more sensitive to the fairness and transparency of AI processes, indicating that experienced users benefit most from developing critical evaluation skills rather than from accumulatting more exposure. Awareness of these individual differences can help students tailor their own preparation priorities rather than adopting a one-size-fits-all approach.

### 5.3 Limitations and directions for future research

Our research has several limitations that point to directions for future work. First, our sample was drawn from Chinese university students, and cultural factors such as attitudes toward AI, career planning norms, and social comparison tendencies may differ across national contexts. While this focus is appropriate given China’s leading role in AI adoption in recruitment, cross-cultural replication would clarify the boundary conditions of these findings. Students from different cultural backgrounds may hold different levels of technology anxiety or social comparison orientation, and future research could investigate whether the pattern of associations within each pathway holds across cultural contexts. Second, AI literacy was measured using self-report scales, which capture perceived rather than objective competence. Future research could incorporate performance-based AI literacy assessments to complement self-report measures. Similarly, AI usage experience was measured with a single indicator of usage frequency; a multi-item scale would allow the depth and breadth of AI experience to be distinguished. Perceived employability was measured with an adapted instrument; although it met the criteria reported in Section 4.1, its equivalence to the source scale has not been established independently. Third, social comparison orientation moderated the AI literacy association but not those of metacognition, NFC, or technology anxiety. This selective pattern is theoretically interpretable, but future research could examine whether alternative boundary conditions—such as growth mindset, tolerance for ambiguity, or AI-specific self-efficacy—prove more consequential for the remaining expectancy-pathway relationships. Fourth, as generative AI interviewers become more interactive and conversational, the nature of both pathways may change: the expectancy process may be influenced by the quality of AI-generated feedback, and the value process may depend on the perceived authenticity of the interaction rather than static transparency disclosures. Future experimental designs that systematically manipulate specific AI interview features—such as transparency levels, feedback provision, or avatar design—could identify which design choices most effectively support each pathway. Finally, participants responded to a described AI interview rather than completing one: no participant underwent algorithmic evaluation or received AI-generated feedback during the study, and 45.6% reported no prior AI interview experience of any kind. The findings therefore speak to associations formed within an imagined AI interview context. While the scenario-based design follows established practices in applicant reactions research (Lievens and Highhouse, 2003; Walker et al., 2013) and ensured a standardized evaluation context, a field or experimental study in which participants actually complete an AI interview, with transparency, feedback, or explanation manipulated, would provide a stronger basis for causal inference; longitudinal field studies tracking students across actual AI interview encounters would likewise complement the present findings and could test whether accumulated experience gradually shifts the psychological basis of career evaluations from competency-based self-appraisal toward process-based organizational appraisal. Two further caveats bear on inference: because the design is cross-sectional and single-wave, all reported paths should be read as associations rather than causal effects; and although common method bias was assessed with five complementary procedures, a purpose-designed marker variable or multi-source, multi-wave data would provide a stronger safeguard than the statistical remedies available here.

In conclusion, this study indicates that university students’ responses to AI video interviews are organized along two partially differentiated pathways with asymmetric antecedent patterns. Preparation for AI-mediated recruitment is widely framed as a matter of technological knowledge. In this sample, employability confidence was associated with self-regulatory, motivational, and affective resources as strongly as with AI literacy, which brings a wider set of capacities within the scope of career preparation. As AI continues to reshape how graduates enter the labor market, understanding and addressing both sides of the employment equation will be essential for universities seeking to prepare students who are not only capable of performing under algorithmic evaluation but also willing to pursue opportunities with organizations that use it.

## Data Availability

The raw data supporting the conclusions of this article will be made available by the authors, without undue reservation.
