# Supplementary material for: When algorithms evaluate people: how AI video interviews relate to employability confidence and organizational attractiveness
Source: Front Psychol. 2026 Sep 25;17:1927892. doi: 10.3389/fpsyg.2026.1927892 (PMC13649998; doi:10.3389/fpsyg.2026.1927892)
Supplement: Supplementary file 1 [file Supplementary_file_1.docx]

**Appendix A. Supplementary analyses**

Manuscript ID 1927892. *When Algorithms Evaluate People: How AI Video Interviews Relate to Employability Confidence and Organizational Attractiveness.*

This appendix reports four analyses that support statements made in the main text: the saturated model behind the dual-pathway claim (Table A1), the nine interactions that were estimated but not hypothesized (Table A2), the relative importance of the four expectancy-pathway predictors (Tables A3 and A4), and the sensitivity of the H7b moderation to how AI experience is operationalized (Table A5). Estimation follows the procedure described in Section 3.3. Estimates are bootstrapped with 10,000 resamples unless a table note states otherwise.

**Table A1. Saturated model: all six antecedents predicting both outcomes**

| **Path** | **β** | **t** | **95% CI** | **Type** |
| --- | --- | --- | --- | --- |
| AIL → PE | 0.188 | 4.40 | [0.106, 0.274] | Hypothesized |
| MC → PE | 0.323 | 7.25 | [0.237, 0.411] | Hypothesized |
| NFC → PE | 0.262 | 5.67 | [0.174, 0.354] | Hypothesized |
| TA → PE | −0.195 | −3.61 | [−0.299, −0.088] | Hypothesized |
| PJ → PE | 0.038 | 0.95 | [−0.038, 0.116] | Cross-path |
| AT → PE | −0.002 | −0.05 | [−0.091, 0.086] | Cross-path |
| PJ → OA | 0.346 | 8.37 | [0.265, 0.428] | Hypothesized |
| AT → OA | 0.260 | 5.85 | [0.169, 0.346] | Hypothesized |
| AIL → OA | −0.045 | −1.09 | [−0.123, 0.042] | Cross-path |
| MC → OA | 0.108 | 2.83 | [0.032, 0.183] | Cross-path |
| NFC → OA | 0.023 | 0.65 | [−0.048, 0.094] | Cross-path |
| TA → OA | −0.243 | −5.33 | [−0.332, −0.154] | Cross-path |

*Note.* N = 520. Gender, age, and education included as controls. 10,000 bootstrap resamples. R² = .581 for perceived employability and .589 for organizational attractiveness; the corresponding values in the hypothesized model are .581 and .541. Admitting the two process perceptions into the perceived employability equation raised R² by less than .001.

**Table A2. Exploratory interactions not covered by H7a or H7b**

| **Interaction** | **DV** | **β** | **t** | **95% CI** | **f²** |
| --- | --- | --- | --- | --- | --- |
| SCO × MC | PE | −0.001 | −0.03 | [−0.070, 0.068] | < .001 |
| SCO × NFC | PE | 0.003 | 0.09 | [−0.065, 0.059] | < .001 |
| SCO × TA | PE | 0.010 | 0.26 | [−0.057, 0.088] | < .001 |
| AI Use × AIL | PE | −0.021 | −0.70 | [−0.077, 0.041] | 0.002 |
| AI Use × MC | PE | −0.029 | −0.78 | [−0.101, 0.043] | 0.002 |
| AI Use × NFC | PE | −0.063 | −1.84 | [−0.126, 0.008] | 0.009 |
| AI Use × TA | PE | 0.091 | 2.94 | [0.025, 0.147] | 0.022 |
| SCO × PJ | OA | −0.001 | −0.03 | [−0.069, 0.065] | < .001 |
| SCO × AT | OA | −0.021 | −0.62 | [−0.084, 0.045] | 0.001 |

*Note.* N = 520. Two-stage moderation with 10,000 bootstrap resamples. These nine interactions were estimated but not hypothesized, and are reported here so that all twelve interactions of the two moderators with the six antecedents are on record. One of the nine reached significance; with nine tests at α = .05 we do not interpret it further.

**Table A3. Relative importance of the four expectancy-pathway predictors**

| **Predictor** | **Relative weight** | **% of R²** | **General dominance** | **% of R²** |
| --- | --- | --- | --- | --- |
| AI literacy | 0.109 | 19.1 | 0.108 | 19.1 |
| Metacognition | 0.172 | 30.3 | 0.171 | 30.1 |
| Need for cognition | 0.156 | 27.4 | 0.155 | 27.3 |
| Technology anxiety | 0.131 | 23.1 | 0.134 | 23.5 |

**Table A4. Bootstrap comparisons of relative weights**

| **Comparison** | **Δ weight** | **95% CI** | **p** |
| --- | --- | --- | --- |
| Metacognition vs. AI literacy | 0.064 | [−0.014, 0.141] | .107 |
| Metacognition vs. need for cognition | 0.016 | [−0.065, 0.098] | .690 |
| Metacognition vs. technology anxiety | 0.041 | [−0.040, 0.123] | .334 |
| Need for cognition vs. AI literacy | 0.047 | [−0.021, 0.118] | .186 |
| Technology anxiety vs. AI literacy | 0.023 | [−0.036, 0.082] | .458 |

*Note.* N = 520. Relative weights follow Johnson (2000) and general dominance weights follow Budescu (1993). Both were computed on the PLS construct scores of the four expectancy-pathway predictors and perceived employability; the four weights sum to the R² of that regression (.568, against .581 for the reported model, which also includes the control variables). Differences between weights were tested against their bootstrap distributions (5,000 resamples); no comparison reached significance. Metacognition held the largest weight in 59.2% of resamples and AI literacy the smallest in 70.6%. The ordering is therefore descriptive, as stated in Section 4.2.

**Table A5. AI experience indicators as moderators of the value pathway**

| **Moderator** | **Interaction** | **β** | **t** | **95% CI** | **f²** |
| --- | --- | --- | --- | --- | --- |
| Two-item composite | PJ × moderator → OA | 0.074 | 2.41 | [0.015, 0.136] | 0.015 |
|  | AT × moderator → OA | 0.070 | 2.26 | [0.009, 0.132] | 0.013 |
| AI usage experience (reported model) | PJ × moderator → OA | 0.085 | 3.11 | [0.030, 0.138] | 0.019 |
|  | AT × moderator → OA | 0.076 | 2.69 | [0.020, 0.132] | 0.015 |
| Previous AI interview experience | PJ × moderator → OA | 0.028 | 0.79 | [−0.038, 0.100] | 0.002 |
|  | AT × moderator → OA | 0.025 | 0.74 | [−0.040, 0.094] | 0.002 |

*Note.* N = 520. PJ = procedural justice; AT = algorithmic transparency; OA = organizational attractiveness. Two-stage moderation with 10,000 bootstrap resamples; each specification re-estimates the full model with the stated moderator. The two indicators correlate at r = .14, and 45.6% of participants reported no previous AI interview. Moderation in the value pathway is carried by AI usage experience: with previous AI interview experience as the sole moderator, neither interaction reaches significance. H7b is therefore specified with AI usage experience alone, and previous AI interview experience is reported as a sample characteristic (Table 1).

**References cited in this appendix**

Budescu, D. V. (1993). Dominance analysis: A new approach to the problem of relative importance of predictors in multiple regression. *Psychological Bulletin, 114*(3), 542–551. https://doi.org/10.1037/0033-2909.114.3.542

Johnson, J. W. (2000). A heuristic method for estimating the relative weight of predictor variables in multiple regression. *Multivariate Behavioral Research, 35*(1), 1–19. https://doi.org/10.1207/S15327906MBR3501_1
